# Prevalence of sexual dysfunction and related factors among diabetes mellitus patients in Southwest Ethiopia

**DOI:** 10.1186/s12902-019-0473-1

**Published:** 2019-12-18

**Authors:** Adane Asefa, Tadesse Nigussie, Andualem Henok, Yitagesu Mamo

**Affiliations:** 1 Department of Public Health, College of Health Science, Mizan- Tepi University, Mizan-Aman, Ethiopia; 2 Department of Pharmacy, College of Health Science, Mizan- Tepi University, Mizan-Aman, Ethiopia

**Keywords:** Diabetes mellitus, Sexual dysfunction, Sexual disorder, Sex disorder

## Abstract

**Background:**

Diabetes mellitus causes multiple medical, psychological and sexual problems in both men and women. Sexual dysfunction is one of those problems that lead to a strong social and psychological problem which adversely affect marital relation and treatment outcome. The issue has not been well studied in Ethiopia; therefore, this study aimed to evaluate the prevalence and factors related to sexual dysfunction in adult patients with diabetes mellitus.

**Methods:**

Facility-based cross-sectional study was conducted among adults with diabetes mellitus on follow-up at diabetic clinics of Mizan-Tepi University Teaching Hospital and Tepi General Hospital. A consecutive sampling technique was employed to select 423 study participants, and data were collected through interviewer-administered questionnaire and patients’ medical chart review. Changes in Sexual Functioning Questionnaire-fourteen items (CSFQ-14) was used to measure sexual dysfunction. Descriptive statistics and binary logistic regression analyses were performed. Two tail tests at α of less 0.05 were used as a level of significance.

**Results:**

A total of 398 diabetic patients were interviewed, with a 94% response rate. The prevalence of sexual dysfunction was 53.3%. Age of above 41 years (AOR: 3.98, 95% CI 2.32–6.85), lack of formal education (AOR: 3.20, 95% CI 1.60–6.39), divorced or widowed (AOR: 5.28, 95% CI 2.35–11.86), type 2 DM (AOR: 4.52, 95% CI 2.17–9.42), depression (AOR: 4.05, 95% CI 2.32–7.10), complications or co-morbidity (AOR: 2.05, 95% CI 1.18–3.58), and not doing physical activity (AOR: 1.62, 95% CI; 1.47–1.77) were significantly associated with sexual dysfunction among diabetes patients.

**Conclusions:**

The prevalence of sexual dysfunction was as high as reports from other studies. Therefore, health care providers should include the issue of sexual health in their routine discussions with adult diabetes mellitus patients. Presence of depression, not doing physical activity and having complications or co-morbidity are modifiable factors associated with sexual dysfunction; therefore, they should be attended during care addressing sexual dysfunction.

## Background

Diabetes mellitus (DM) is one of the common chronic diseases in the world. About 422 million people were affected in 2014; this figure shows an increasing trend when compared with that of 1980, which was about 108 million [1]. International Diabetic Association (IDA) report of 2015 estimated that the number of people with diabetes could be increased to 642 million by 2040. The report also shows Ethiopia could be accounted for about 2,567,900 cases of diabetes [2]. According to World Health Organization (WHO) report, DM caused about 1.5 million deaths in 2012 [1]. Also, diabetes and its complications bring substantial economic loss to people with diabetes and their families, and health systems and national economies through direct medical costs and loss of work and wages [1].

Sexual dysfunction frequently occurs among DM patients [3]. Studies reported the high prevalence, and earlier onset of sexual dysfunction among diabetic men when compared to non-diabetic [3–5]. Similarly, the study conducted in northern Ethiopia showed the prevalence of erectile dysfunction among DM patients was 69% [6]. Also, another study has documented low sexual desire, lack of sexual satisfaction, low vaginal lubrication and orgasmic dysfunction among women with DM [7].

Strong physical, social and psychological problems are associated with sexual dysfunction. For instance, erectile disorder and premature ejaculation are related to anxiety. Moreover, low sexual satisfaction, sadness, low self-esteem, distress, and depression are very common among men who are suffering from sexual dysfunction. In women, arousal, orgasmic and enjoyment problems are associated with anxiety and depression [8, 9]. Furthermore, sexual dysfunction imposes a challenge to partner relationships. It results in less marital satisfaction, emotional stress, less communication, and difficulty in resolving problems, and finally, it may lead to divorce [10, 11]. Moreover, patient self-care behavior decreases when there is conflict in marital relationships, and this, in turn, results in poor glycemic control [12].

Few studies were conducted on sexual dysfunction among diabetic patients in Ethiopia, and they focused on erectile dysfunction only [6, 13]. Moreover, open talk about sexual related issue is a taboo in Ethiopia due to the societal norm. Because of this, most patients do not freely discuss sexual problems with their doctor or partner. As a result, sexual disorder is considered as under-recognized and under-treated disorders in the country. Therefore, this study aimed to evaluate the prevalence and factors related to sexual dysfunction in adult patients with diabetes mellitus.

## Methods

### Study design and setting

A facility-based cross-sectional study was conducted from July 01–31/ 2018 among diabetes mellitus patients on follow-up at diabetic clinics of Mizan Tepi University Teaching Hospital (MTUTH) and Tepi General Hospital (TGH). MTUTH and TGH are located in Southwest Ethiopia at 585 km and 611 km from Addis Ababa respectively. MTUTH is found in Mizan-Aman town. The hospital provides service for the population that comes from Bench-Maji, Sheka and Kafa Zones and some parts of Gembella regional state. It has a bed capacity of 209 and has over 400 staff members. TGH located in Tepi town, and it provides service for people who come from Shaka, Kafa and Majang Zones.

### Study population and eligibility

Adult diabetic patients who had been on follow up at diabetic clinics of MTUTH and TGH were a source population. The study population was a sample of adult DM patients who were on follow up during the study period at the diabetic clinics of MTUTH and TGH, and who fulfilled eligibility criteria. Patients who were mentally impaired and unable to give information and/or had sexual disorder before the onset of diabetes mellitus were excluded. Also, females aged above 50 years, and/or those who had history of pelvic surgery were excluded.

### Sample size and sampling procedure

The sample size was determined manually using a single population proportion formula ($n=\frac{{\left({z}_{\alpha /2}\right)}^2p\left(1-p\right)}{d^2}$) [14]; based on the assumptions of 95% confidence level, 5% margin of error and a 50% proportion of sexual dysfunction. The prevalence of 50% was taken because there was no similar study done in Ethiopia previously. After adding 10% contingency for non-response, the final sample size for the study was 423. Based on the total number of patients on follow up at each hospital, the sample size was proportionally allocated to the two study settings. Accordingly, 279 samples were allocated to MTUTH and 144 samples to TGH. The study participants were recruited during their regular medical follow-up period consecutively based on their arrival at DM clinics of each Hospital.

### Data collection tools and procedure

Changes in Sexual Functioning Questionnaire-fourteen items (CSFQ-14) was adopted and used to measure sexual dysfunction [15]. The questionnaire to assess socio-demographic and behavioral related factors was developed by reviewing different literature. Also, data extraction checklist was prepared to collect data related to medical history. The tool was translated to a local language, “Amharic” then back to English, to ensure its consistency. Then, it was pre-tested on 5% of the total sample size at Chenna Hospital before the actual data collection to evaluate readability, understandability, completeness, and reliability of the questionnaire, and modified accordingly. Internal consistency for CSFQ-14 was checked and demonstrated Cronbach’s alpha of 0.72 and 0.81 for women and men respectively. Finally, the Amharic version was used to collect the data. Trained personnel collected the data through face-to-face interview method. The most recent recorded information related to medical condition were extracted from patient medical chart. Due to the sensitivity of the issue, female data collectors were assigned to collect data from the female participants whereas male data collectors collected data from male participants.

### Study variables

The dependent variable of the study was sexual dysfunction. The independent variables were socio-demographic information (age, sex, religion, marital status, educational status, ethnicity and place of residence), factors related to medical condition (types of DM, duration of treatment, glycemic control, complication or co-morbidity and depression) and behavioral factors (physical activity, adherence to antidiabetic medication, alcohol, tobacco and khat use).

### Measurements

Sexual dysfunction was measured using CSFQ-14, a standardized tool used to screen sexual activities. The CSFQ-14 has 14 separate items for males and females to assess sexual function. All items were answered on five Likert scales. The responses were summed up to give a total score of 14–70. The score at or below 47 for males and 41 for females indicates the presence of global sexual dysfunction. The tool also measures five types of sexual dysfunction (pleasure, desire/frequency, desire/interest, arousal/erection and orgasm/ejaculation). Scores of less than or equal to 6,9,4,3, and 13 indicate sexual desire/frequency, sexual desire /interest, sexual pleasure, sexual arousal/excitement, and sexual orgasm/completion dysfunctions respectively in males. In females, scores at or below 6, 9, 4, 12, and 11 show the presence of sexual desire frequency, sexual desire/interest, sexual pleasure, sexual arousal/excitement, and sexual orgasm/completion dysfunctions respectively.

Glycemic control was assessed using more recent fasting blood sugar (FBS) measurements’ of three months. Patients were considered in good glycemic control range if the average FBS level was ≤130 mg/dL, and poor glycemic control if it was > 130 mg/dL. Depression symptoms in the past 14 days of the survey were measured using validated patient’s health questionnaire-nine (PHQ-9), which is a validated tool [16]. The PHQ- 9 has nine items, each item having 4-point response scales; ‘not at all’ (0), ‘various days’ [1], ‘more than half of the days’ [2] and ‘nearly every day’ [3]. Summing all these possible, the total score range from 0 to 27 points. The score of 0–4 indicates no or minimal depression, 5–9 is mild depression, 10–14 is moderate depression, 15–19 moderately severe depression and 20–27 severe depression. Overall cutoff ≥10 was used to indicate depression. Data related to complication or comorbidity was obtained from patients’ medical charts based on physician diagnosis. If patients had at least one of these conditions; nephropathy, retinopathy, diabetic neuropathy, diabetic foot ulcer, cardiac disease or hypertension they were considered as they had complication or co-morbidity otherwise no. Complication and co-morbidity were merged together because it is very difficult to differentiate them from secondary data if these conditions were occurred secondary to DM or happened as co-morbidity. Duration on anti-diabetic treatment was assessed based on patient self-report of the years elapsed since they started the medication.

Adherence to physical activity was measured based on the participants’ responses. Patients who had been regularly engaged in moderate (like slow walking or dancing) to vigorous-intensity exercise (like fast walking or running) at least 2 days per week were classified as adherent to physical activity. We assessed adherence to anti-diabetic medication in the past seven days of survey and respondents were considered adherent if they took all anti-diabetic medication as per the prescription of a physician. The respondents were categorized as substance users if they reported consumption of any of the following substances in any amount in the past 12 months; had drunk common alcohols in the area such as beer, wine or local alcohols like ‘Areke’, ‘Teji’or ‘Katikala’; smoked tobacco, chewed ‘khat.

### Data processing and analysis

Data were cleaned, coded and entered into Epi data version 3.1 and exported to SPSS version 21 for analysis. Data were explored for unexpected values and outliers and compiled for final analysis. Descriptive statistics such as proportion, mean and standard deviation were computed for categorical and continuous variables as supposed necessary. Bivariate binary logistic regression analyses were done for all independent variables and variables with a *p*-value less than 0.25 were considered as candidates for the multivariable model. Finally, multivariable binary logistic regression analysis was done, and a p-value of ≤0.05 considered as statistically significant. The strength of association was measured using odds ratio at 95% confidence level. Model fitness was evaluated using the Hosmer-Lemeshow test. In the final model, the extent of multicollinearity was measured using the variance inflation factor (VIF) which was found to be within a tolerable range (less than 10).

### Ethical consideration

Ethical clearance was obtained from the Institutional Review Board (IRB) of Mizan-Tepi University (Ref No: CHS/205/2018). Data were collected after obtaining written informed consent from all respondents. All the information gathered was kept confidential.

## Results

### Socio-demographic characteristics

Among planned 423 planned samples, 398 (94%) were successfully involved in the study. The mean age of the respondents was 41.76 (±9.00) years and 40.20% were in the age range of 41 to 50 years. Out of the total respondents, 64.3% were male, 35.4% were protestant Christian, 43.2% had attended secondary and above education, 64% were married, and 71.4% of respondents were urban residents (Table 1).

**Table 1:** Socio-demographic characteristics of the respondents MTUTH and TGH southwest Ethiopia, July 2018

Variables	Categories	Frequency	Percent
Age	18–30	54	13.6
	31–40	117	29.4
	41–50	160	40.2
	51+	67	16.8
Sex	Male	256	64.3
	Female	142	35.7
Religion	Orthodox	136	34.2
	Protestants	141	35.4
	Muslim	96	24.1
	Others	25	6.3
Marital status	Married	263	66.1
	Single	49	12.3
	Divorced /widowed	86	21.6
Ethnicity	Kaficho	106	26.6
	Amhara	101	25.4
	Bench	60	15.1
	Shakacho	50	12.6
	Others	80	20.1
Educational status	No education	106	26.6
	Primary	120	30.2
	Secondary and above	172	43.2
Residence	Urban	284	71.4
	Rural	114	28.6

### Behavioral and medical-related factors

**Fig. 1:**
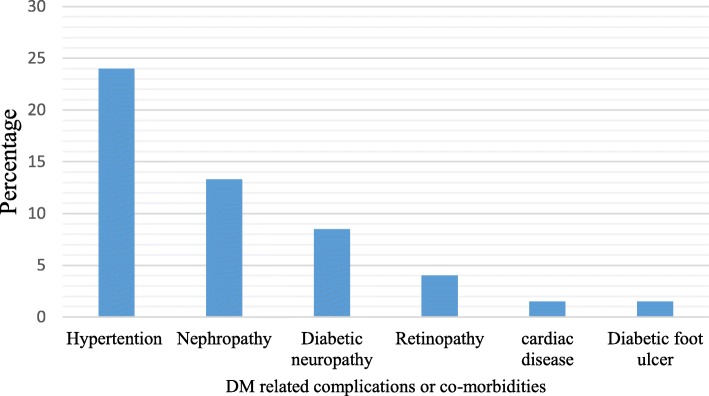
DM-related complications among respondents, MTUTH and Tepi- General Hospital southwest Ethiopia July 2018 (*n* = 398)

Nearly two-thirds (67.6%) of study participants were engaged in moderate to vigorous physical activity. One hundred fifty-eight (39.7%) respondents had history of substance use in the past 12 months. The substance commonly used was alcohol (20.9%). Seventy-four percent of the respondents adhered to antidiabetic medication in the past 7 days before the interview (Table 2). Out of 398 respondents, 318 (79.9%) were Type 2 Diabetes Mellitus (T2DM) patients. Based on the American Diabetic Association (ADA) guideline recommendation, 87.9% of the patients were in poor control glycaemic range (FBS > 130 mg/dL). The prevalence of depression among study participants was 36.9% (Table 3). Moreover, 39% of patients were experienced complications related to DM and the common complication was hypertension (24.3%) (Fig. 1).

**Table 2:** Behavioral related factors of respondents, MTUTH and TGH southwest Ethiopia, July 2018 (*n* = 398)

Variables	Categories	Frequency	Percent
Physical exercise	Yes	269	67.6
Substance use	Yes	158	39.7
Adherence to anti-diabetic medication	Adherent	293	73.6
	Not Adherent	105	24.4
Alcohol	Yes	83	20.9
Khat	Yes	54	13.8
Tobacco	Yes	35	9

**Table 3:** Medical condition of the respondents, MTUTH and TGH Southwest Ethiopia, July 2018 (*n* = 398)

Variables	Categories	Frequency	Percent
Type of DM	T1DM	80	20.1
	T2DM	318	79.9
Duration of treatment	< 5 years	299	75.1
	> = 5 years	99	24.9
Glycemic control	Poor	350	87.9
	Good	48	12.1
Complication or co-morbidity	Yes	212	53.3
	No	186	46.7
Depression	Yes	147	36.9
	No	251	63.1

Poor: FBS > 130 mg/dL, Good: FBS ≤130 mg/dL

Complication or co-morbidity (nephropathy, retinopathy, diabetic neuropathy, diabetic foot ulcer, cardiac disease and/or hypertension)

### Prevalence of sexual dysfunction

**Fig. 2:**
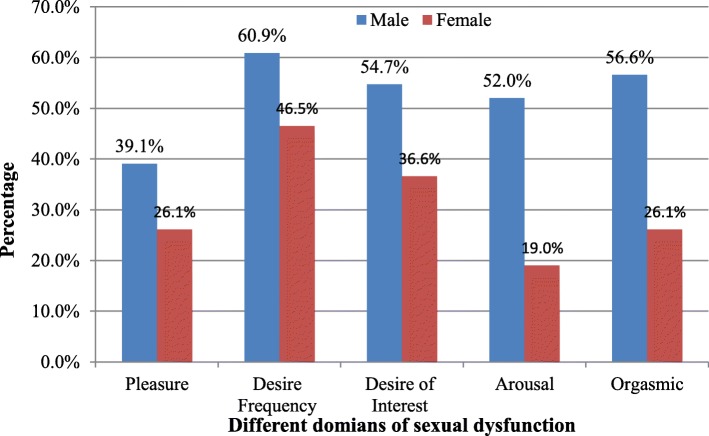
Prevalence of different domain of sexual dysfunction among male and female, MTUTH and Tepi General Hospital southwest Ethiopia, July 2018 (Males = 256, Females = 142)

The prevalence of global sexual dysfunction among study participants was 53.3% (95% CI: 48.35–58.25%). Gender wise, the prevalence of global sexual dysfunction was 52% (95% CI: 46–58%) among males and 56.6% (95% CI: 47–64%) among females. The most prevalent dysfunction among men was frequency of sexual desire disorder (60.9%) followed by orgasmic dysfunction (56.6%). Among women, frequency of sexual desire disorder was the most common sexual dysfunction (46.5%), followed by desire (interest) dysfunction (36.6%) (Fig. 2).

### Factors associated with sexual dysfunction

In bivariate binary logistic regression analyses, age, marital status, educational status, physical activity, substance use, adherence to anti-DM medication, type of DM, duration of treatment, complication of DM or comorbidity and presence of depression had a *p*-value of ≤0.25; hence, they were candidates for a multivariable model. However, sex, religion, ethnicity, and duration of treatment were not significant at *p*-value of ≤0.25; therefore, they were excluded from multivariable analysis. In the multivariable binary logistic model, age of respondents, educational status, marital status, types of diabetes mellitus, complication or comorbidity, the presence of depression and physical activity were significantly associated (*p* < 0.05) with sexual dysfunction.

Based on adjusted odds ratio, DM patients in the age range of above 41 years had 3.98 times higher odds of sexual dysfunction as compared to those in the age range of less than or equal to 41 years (AOR = 3.98; 95% CI: 2.32–6.85). The odds of sexual dysfunction was 3.2 times higher for patients who did not attend education in reference to those who attended secondary school or above education (AOR = 3.20; 95% CI: 2.32–6.85). However, there was no statistically significant difference in odds of sexual dysfunction between patients who attended and secondary school or above (AOR =0.46; 95%:0.20–1.00). Participants who did not involve in physical activity had 1.62 (AOR = 1.62; 95% CI: 1.47–1.77) times higher odds of sexual dysfunction than the counterpart. With reference to Type 1 Diabetes Miletus (T1DM), the odds of sexual dysfunction among T2DM patients were 4.52 times higher (AOR =4.52: 95% CI: 2.17–9.42). The sexual dysfunction among patients who had complication or comorbidity was 2.05 times (AOR = 2.05; 95% CI: 1.18–3.58) higher when compared to those who didn’t have such history. The odds of sexual dysfunction was 4 times higher for diabetic patients who had symptoms of depression as compared to those who had no depression (AOR = 4.05; 95% CI: 2.32–7.10) (Table 4).

**Table 4:** Factors associated with sexual dysfunction among respondents, MTUTH and TGH Southwest Ethiopia July, 2018

Variables	Categories	Sexual dysfunction		COR (95% CI)	AOR (95% CI)	*P*-value
		Yes (%)	No (%)			
Age group	<= 41	70(38)	144(62)	1	1	
	> 41	142(66.4)	72(33.6)	3.21(2.13–4.85)	3.98 (2.32–6.85)	0.00
Educational status	No education	60 (56.6)	46(43.4)	3.81(2.31–6.27)	3.20 (1.60–6.39)	0.00
	Primary	85(70.8)	35(29.2)	2.04(1.25–3.34)	0.46 (0.20–1.00)	0.318
	Secondary and above	67(39)	105(61)	1	1	
Exercise	Yes	130(48.3)	139(51.7)	1	1	
	No	82(63.6)	47(36.4)	1.87 (1.21–2.87)	1.62 (1.47–1.77)	0.037
Marital status	Married	114(43.3)	149(56.7)	1	1	0.007
	Single	25(51)	24(49)	1.36 (0.74–2.50)	1.59 (0.67–3.78)	0.78
	Divorced and widowed	73(84.9)	13(15.1)	7.34(3.88–13.89)	5.28(2.35–11.86)	0.02
Types of DM	T1DM	25(31.3)	55(68.8)	1	1	
	T2DM	187(58.8)	131(41.2)	3.14(1.86–5.30)	4.52 (2.17–9.42)	0.00
Complication/co-morbidity	Yes	97(62.2)	115(47.5)	1.82(1.21–2.74)	2.05(1.18–3.58)	0.01
	No	59(37.8)	127(52.5)	1	1	
Depression	Yes	102(69.4)	45(3.6)	2.91(1.89–4.67)	4.05(2.32–7.10)	0.00
	No	110(43.8)	141(56.2)	1	1	

## Discussion

The study revealed that the prevalence of sexual dysfunction among diabetes mellitus patients was 53.3%. This magnitude is similar to the study conducted among diabetic patients in Tanzania [17]. Other studies also reported high prevalence of sexual dysfunction among DM patients [18, 19]. But, this finding is lower compared with the study done in Iran [20], Tigray [21] and Felege Hiwot Referral Hospital, Northern Ethiopia [13]. This variation might be due to differences in the socio-cultural related factors of study participants and the tool used to measure sexual dysfunction in different studies. For instance, the studies conducted in Tigray and Felege Hiwot Referral Hospital used the International Index of Erectile Function (IIEF), and the study conducted in Iran used IIEF and Female Sexual Function Index (FSFI). The high prevalence of sexual disorders amongst DM patients could be due to prolonged hyperglycemia that causes impairment of sexual functions by causing atherosclerosis, diabetic neuropathy, diabetes-induced endothelial dysfunction and endocrinological changes [19, 22]. Yet, the high prevalence of sexual dysfunction in this study might be due to lack of control for some medical, psychological and drug-related factors that can affect sexual function.

In this study higher age were associated with increased odds of sexual dysfunction in DM patients. A similar finding was published by a study conducted at Felege Hiwot Referral Hospital [13]. Furthermore, the finding of this study is consistent with a report from other similar studies [17, 20]. The increased risk of sexual dysfunction at older age, might be due to advanced age is correlated with long duration of treatment, increased DM related complications and/or other co-morbidities, poor glycemic control, change in hormonal function and BMI which can affect sexual function.

Similar to a report from another study [23], patients who did not attend education had a higher risk of having sexual dysfunction. This might be due to uneducated people had limited get access to health information; hence, they may not adhere to behaviors that delay or prevent DM complications. Besides, they may unaware of the disorder and its treatment; thus, they may not seek treatment for it. Moreover, this might be due to not attending education in most of the case associated with unemployment and financial insecurities that can result in inability to pay for medical cost, and having poorer nutritional choices, which can cause poor glycemic control and finally leads to sexual dysfunction or other complications.

In this study, participants who were widowed or divorced had a higher risk of sexual dysfunction when compared to those in the marital union. This finding is supported by a study done at Amanuel Mental Specialized Hospital [24]. This might be due to individuals in the marital union can get positive benefits such as high social and psychological support that improve adherence to antidiabetic medications. This, in turn, leads to good treatment response, and delay complications of DM. In addition, divorced or widowed individuals probably have decreased frequency of sexual desire due to the loss of their beloved partner. On the other hand, sexual dysfunction itself imposes a challenge on partner relationships and might result in divorce.

The study also reveals that patients with T2DM were at an increased risk of experiencing SD compared to patients with T1DM. This finding is in line with a study conducted in Iran and Tanzania [20, 25]. Also, the finding is consistent with reports from other studies [6, 26]. Diabetic patients who had complication or co-morbidity were twice more likely to have sexual dysfunction when compared to those who did not have complication or co-morbidity. The finding is consistent with reports from different similar studies [7, 27–30]. This could be due to complication related to DM and co-morbidity can contribute to the pathogenesis of sexual dysfunctions. For instance, endocrine-related complication can lead to hypogonadism. Another study reported that sexual dysfunction is a marker for the development of chronic conditions such as DM and coronary artery disease [29]. These findings suggest that diabetic complications may play an important role in decreasing sexual function, and hence prevention of diabetic complications may be help to prevent sexual dysfunction in a patient with diabetes.

The study indicated a strong association between sexual dysfunction and depression. This result is supported by a study done in Iran [24]. Likewise, other studies also revealed an increased risk of sexual disorders among diabetic patients with depressive symptoms [31, 32]. On the other way, a study also showed an increased risk of depression among patients with chronic diseases [33]. A meta-analysis also confirmed a bidirectional association between depression and sexual dysfunctions [34].

In this study, adherence to exercise was associated with decreased risk of sexual dysfunction among DM patients. This is in line with a study conducted in Ghana [35]. Similarly, another study showed that physical activity is a significant predictor of erectile dysfunction [36]. This could be due to exercise is a key aspect of a healthy lifestyle that might benefit DM patients to prevent high blood glucose levels and DM-related complications. Additionally, exercise enhances sexual satisfaction indirectly through restoring autonomic nerve flexibility which in turn helps to improve cardiovascular health and mood [37].

### Limitation

The study addressed the issue that was not well studied in Ethiopia. However, due to the cross-sectional nature of our study, we cannot be sure of the temporal relationship between different factors and sexual dysfunction. Furthermore, the study was done on a culturally sensitive and embarrassing issue, therefore, social desirability bias could not be ruled out. As a result, patients may under-report the problem. In addition, since the study was conducted among patients in hospitals, the finding may not represent patients on follow-up in other settings.

## Conclusion

The prevalence of sexual dysfunction was as high as reports from other studies. Therefore, health care providers should include the issue of sexual health in their routine discussions with diabetes patients. Presence of depression, not doing physical activity and DM complication and/or comorbidity are modifiable factors associated with sexual dysfunction; therefore, they should be attended during care addressing sexual dysfunction.

## Data Availability

The datasets used and analyzed during the current study are available from the corresponding author on reasonable request.

## Abbreviations

ADA: American Diabetic Association
AOR: Adjusted Odds Ratio
CO: Crude Odd Ratio
CSFQ: Change in Sexual Functioning Questionnaire
DM: Diabetes Mellitus
MTUTH: Mizan Tepi University Teaching Hospital
PHQ: Patient Health Questionnaire
SD: Sexual Dysfunction
T1DM: Type 1 Diabetes mellitus
T2DM: Type 2 Diabetes mellitus
TGH: Tepi General Hospital
WHO: World Health Organization
